# The humoral immune response is essential for successful vaccine protection against paratuberculosis in sheep

**DOI:** 10.1186/s12917-019-1972-z

**Published:** 2019-07-02

**Authors:** Hannah B. Pooley, Douglas J. Begg, Karren M. Plain, Richard J. Whittington, Auriol C. Purdie, Kumudika de Silva

**Affiliations:** Sydney School of Veterinary Science, Faculty of Science, The University of Sydney, Private Bag 4003, Narellan, Camden, NSW 2567 Australia

**Keywords:** Antibody, Paratuberculosis, Vaccination, Sheep, Humoral immunity, Ileum, ELISA, Gene expression, Infection

## Abstract

**Background:**

The role played by the humoral immune response in animals vaccinated against a mycobacterial disease such as paratuberculosis, is not well understood. Sheep vaccinated against *Mycobacterium avium subsp. paratuberculosis* (MAP) can still become infected and in some cases succumb to clinical disease. The strength and location of the humoral immune response following vaccination could contribute to the ability of sheep to clear MAP infection. We examined the peripheral antibody response along with the localised humoral response at the site of paratuberculosis infection, the ileum, to better understand how this contributes to MAP infection of sheep following vaccination and exposure.

**Results:**

Through assessing MAP specific serum IgG1 and IgG levels we show that the timing and strength of the humoral immune response directly relates to prevention of infection following vaccination. Vaccinated sheep that subsequently became infected had significantly reduced levels of MAP specific serum IgG1 early after vaccination. In contrast, vaccinated sheep that did not subsequently become infected had significantly elevated MAP specific serum IgG1 following vaccination. Furthermore, at 12 months post MAP exposure, vaccinated and subsequently uninfected sheep had downregulated expression of genes related to the humoral response in contrast to vaccinated infected sheep where expression levels were upregulated.

**Conclusions:**

The timing and strength of the humoral immune response following vaccination against paratuberculosis in sheep directly relates to subsequent infection status. An initial strong IgG1 response following vaccination was crucial to prevent infection. Additionally, vaccinated uninfected sheep were able to modulate that response following apparent MAP clearance, unlike vaccinated infected animals where there was apparent dysregulation of the humoral response, which is associated with progression to clinical disease.

## Background

Humoral immunity is believed to play a role in the protective response against intracellular mycobacterial pathogens, such as *Mycobacterium avium subsp. paratuberculosis* (MAP), the causative agent of paratuberculosis in ruminants [1, 2]. The pathogenesis of paratuberculosis was traditionally characterised by an increase in the humoral response measured through antibody production at the end stages of disease [3, 4]. The switch from an initial dominant cell mediated immune (CMI) response to a humoral response is often thought to signify a breakdown of disease control by the host and progression to clinical disease [5]. While it is undisputed that Interferon gamma (IFNγ) production is essential for overcoming mycobacterial infection [6], the pattern of a protective immune response to MAP infection is actually not so clear [7], with some studies showing MAP-specific antibody responses in sheep as early as two weeks post exposure (wpe) [8] and occurring at the same time as an IFNγ response [9].

The protection provided by vaccines against mycobacterial pathogens, particularly MAP, is often incomplete [10–12]. Sheep and cattle vaccinated against MAP have reduced incidence of clinical disease and faecal shedding, however commercial vaccines fail to prevent infection in all animals [13–15]. Understanding the mechanisms behind how some vaccinated animals successfully clear infection when others do not, would allow development of new vaccines to specifically target a protective immune response in all animals. Traditional markers to assess vaccine efficacy such as IFNγ and total antibody response in isolation are not able to differentiate between animals protected by vaccination and those that are not [16]. Therefore, there is a need to explore alternate or additional markers of vaccine protection to truly understand a protective vaccine response. To this point, most work on correlates of vaccine-induced protection against paratuberculosis has focused on the CMI response, however recent studies have suggested a role for B cells as well [16, 17].

B cells are pivotal in the activation and modulation of both CMI and humoral immune responses [18, 19]. B cells function as antigen presenting cells but also produce antibodies enabling immune complexes that can regulate the function of effector cells such as macrophages [17, 20–23]. In ruminants, the proliferative capacity of peripheral B cells is reduced in animals where vaccination fails to provide protection against MAP [16]. This response was noted as early as 13 weeks’ post MAP exposure. Additionally, a study by Begg and Griffin [24], found significantly higher percentages of B cells in the gut of vaccinated animals that survived MAP challenge compared to diseased animals. Therefore, although peripheral B cells may be functionally impaired, the humoral response at the site of infection might be more important to vaccine-induced protection.

The activity and survival of B cells at the site of infection has been examined in relation to disease progression for mycobacterial infections, but not in response to vaccination. B cells in the tissue can be activated by several different mechanisms, including direct antigen contact, ligation of the CD40 receptor by T cells and binding of B cell activating factor (BAFF) [17]. CD40 ligand (CD40L) deficiency predisposes humans to opportunistic infections by intracellular bacteria [25] and can be correlated with severe tuberculosis in macaques [26]. In contrast, the expression of BAFF by circulating populations of CD4^+^ T cells is associated with active tuberculosis [27]. Other indicators of the humoral immune response, including B cell surface markers (CD81), cytokines that promote B cell survival (*MIFF*) and transcription factors (*JUN*), have all been implicated in the progression or prevention of mycobacterial infections [28–30]. These apparent contradictions suggest that detailed examination of B cell functionality and the humoral response at the site of infection is required. Furthermore, the ability to understand whether the humoral response does provide vaccine-elicited protection against MAP requires an understanding of the host’s ability to successfully mount this response at the site of infection.

We hypothesise that differences in B cell functionality may be correlated with infection status following exposure to MAP in vaccinated sheep. Therefore, we characterised the role of humoral immunity in sheep that were vaccinated and then became infected compared to vaccinated sheep that did not become infected. We assessed MAP-specific IgG1 and IgG levels and compared these with other aspects of B cell functionality, such as cell survival, differentiation, activation and receptor signalling, by gene expression in the gut tissues of these animals.

## Results

### Animal trial

Infection outcome in vaccinated and non-vaccinated sheep at the conclusion of the animal trial was determined by culture of live MAP from the intestine (Table 1.). In the vaccinated animals exposed to MAP, two were found to be infected with live bacteria at 52 wpe and 18 had no culturable MAP at this time. In contrast, in the non-vaccinated sheep, 10 were found to be infected with MAP and 10 were uninfected at 52 wpe.

**Table 1:** Numbers of animals included in IgG1 and tissue gene expression analyses

Treatment	Infection status	n for ELISA	n for gene expression
Vaccination	control	5	3
	uninfected	18	4
	infected	2	2
Non-vaccination	control	5	3
	uninfected	10	3
	infected	10	3

**Fig. 1:**
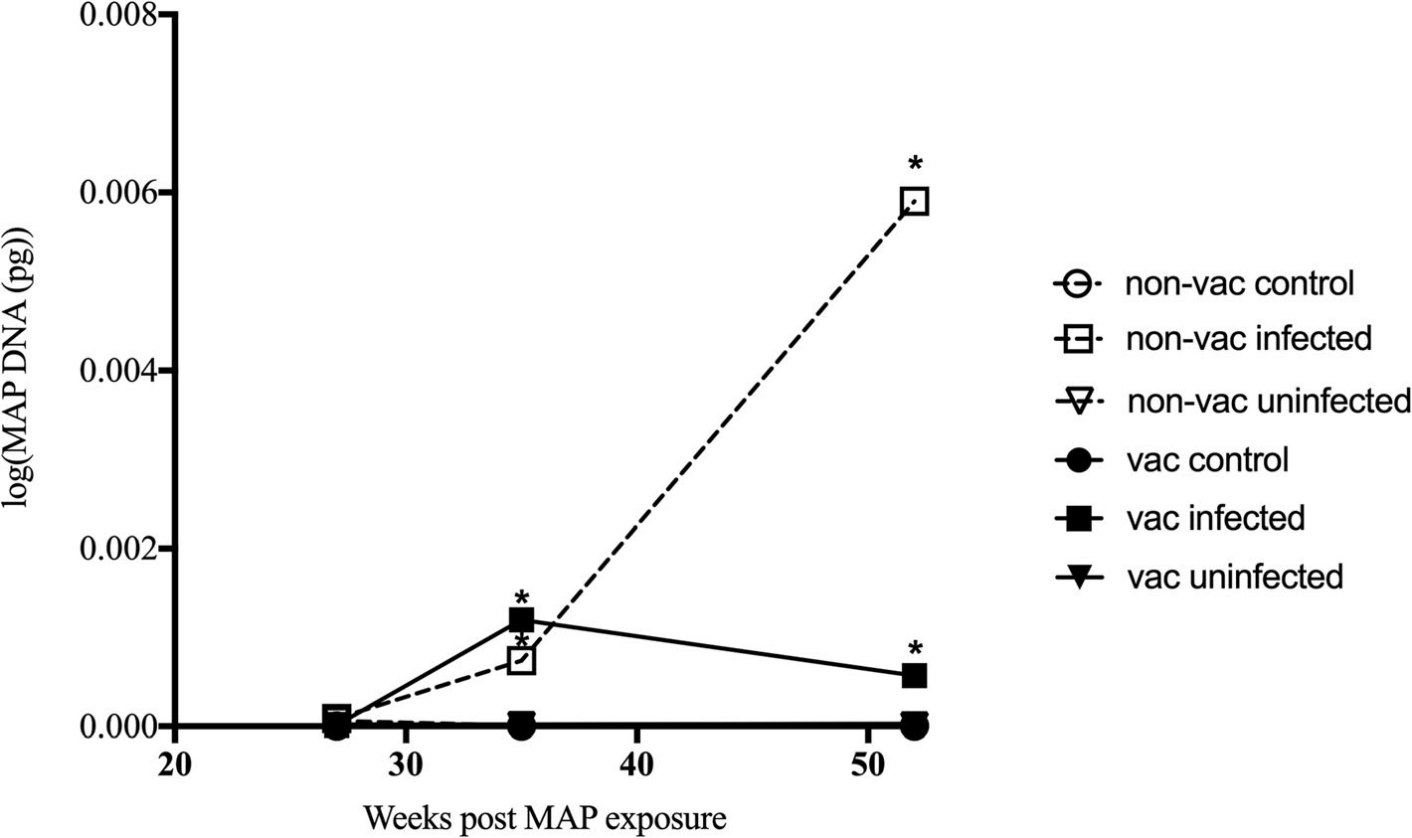
Quantity of MAP DNA shed in the faeces. Gudair™ vaccinated (vac) (6 weeks prior to MAP exposure) and non-vaccinated (non-vac) sheep were either exposed or left unexposed (control) to MAP. MAP exposed sheep were grouped based on infection status (infected and uninfected), determined by tissue culture at necropsy. The quantity (pg) of MAP DNA shed in the faeces was determined by direct faecal PCR at 3 timepoints throughout the trial. * denotes groups significantly different to all other groups not marked with an asterisk (*p* < 0.05)

Vaccinated and non-vaccinated infected sheep shed significantly more MAP in the faeces from 35 weeks post MAP exposure till the end of the trial, compared to both uninfected and control animals (*p* < 0.05) (Fig. 1).

### Serum antibody

**Fig. 2:**
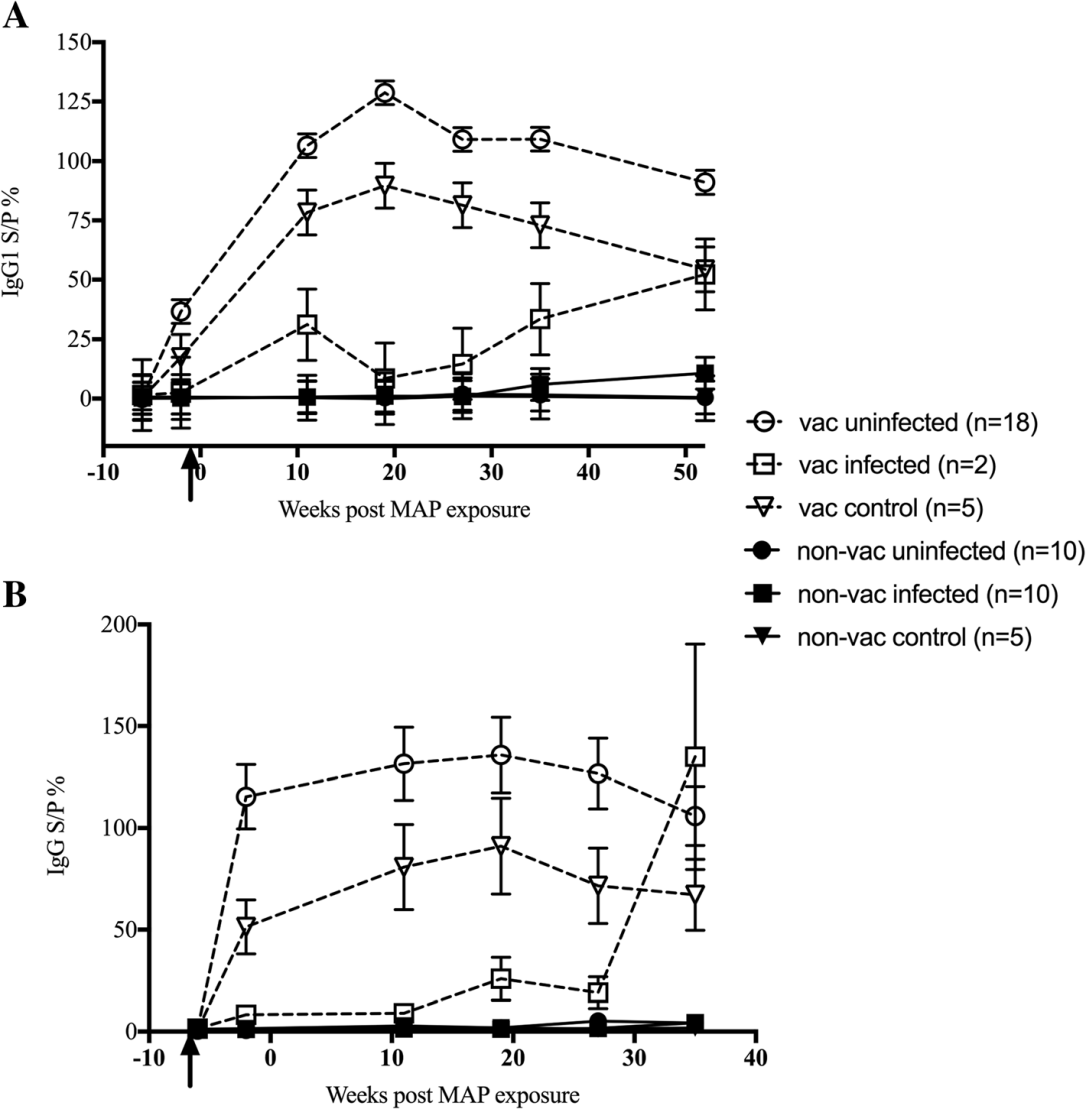
MAP-specific serum antibody response in sheep. Gudair™ vaccinated (vac) (6 weeks prior to MAP exposure) and non-vaccinated (non-vac) sheep were either exposed or left unexposed (control) to MAP. MAP exposed sheep were grouped based on infection status (infected and uninfected), determined by tissue culture at necropsy. MAP-specific IgG1 (**a**) and MAP-specific IgG (**b**) levels in serum were determined by ELISA. Data presented are the predicted mean and standard error, from the linear mixed model analysis. The arrow indicates the timepoint of vaccination. The animal number in each group is denoted in the legend

Vaccination significantly enhanced MAP-specific IgG1 levels in serum of sheep (*p* < 0.001) (Fig. 2a). In the vaccinated exposed animals, serum MAP-specific IgG1 was significantly higher in the cohort found to be uninfected at necropsy, compared to the infected animals. This polarised response was first evident prior to MAP exposure, as early as 1-month post vaccination (*p* < 0.05). Serum MAP-specific IgG1 peaked in the vaccinated uninfected animals at 19 wpe and then tended to wane until the final sampling at 52 wpe. In contrast, the vaccinated infected animals had significantly lower levels of MAP-specific IgG1 (*p* < 0.05). The IgG1 response over time was also dissimilar to the vaccinated uninfected sheep, with the vaccinated infected animal’s MAP-specific IgG1 peaking at 11 wpe, decreased at 19 weeks and then increasing until the final sampling at 52 wpe.

Non-vaccinated animals had very low levels of MAP-specific IgG1 in serum compared to vaccinates. There were no significant differences between the non-vaccinated controls and the two non-vaccinated exposed groups (infected or uninfected) at any time point. However, in the non-vaccinated infected group, similar to the vaccinated infected sheep, MAP-specific IgG1 levels in serum tended to increase at the final sampling time point.

A similar pattern to the serum MAP-specific IgG1 response was also seen in the MAP-specific IgG data (Fig. 2b). Vaccinated animals had significantly greater MAP-specific IgG levels than non-vaccinates (*p* < 0.001). As with the MAP-specific IgG1, vaccinated uninfected sheep had higher MAP-specific IgG levels, which remained high throughout the animal trial. Interestingly, the vaccinated infected sheep had very low MAP-specific IgG levels that were not significantly different from the non-vaccinated sheep, until 11 wpe. At 19 wpe, MAP-specific IgG levels began to increase in these vaccinated infected animals, with a rapid increase from 27 to 35 wpe.

**Fig. 3:**
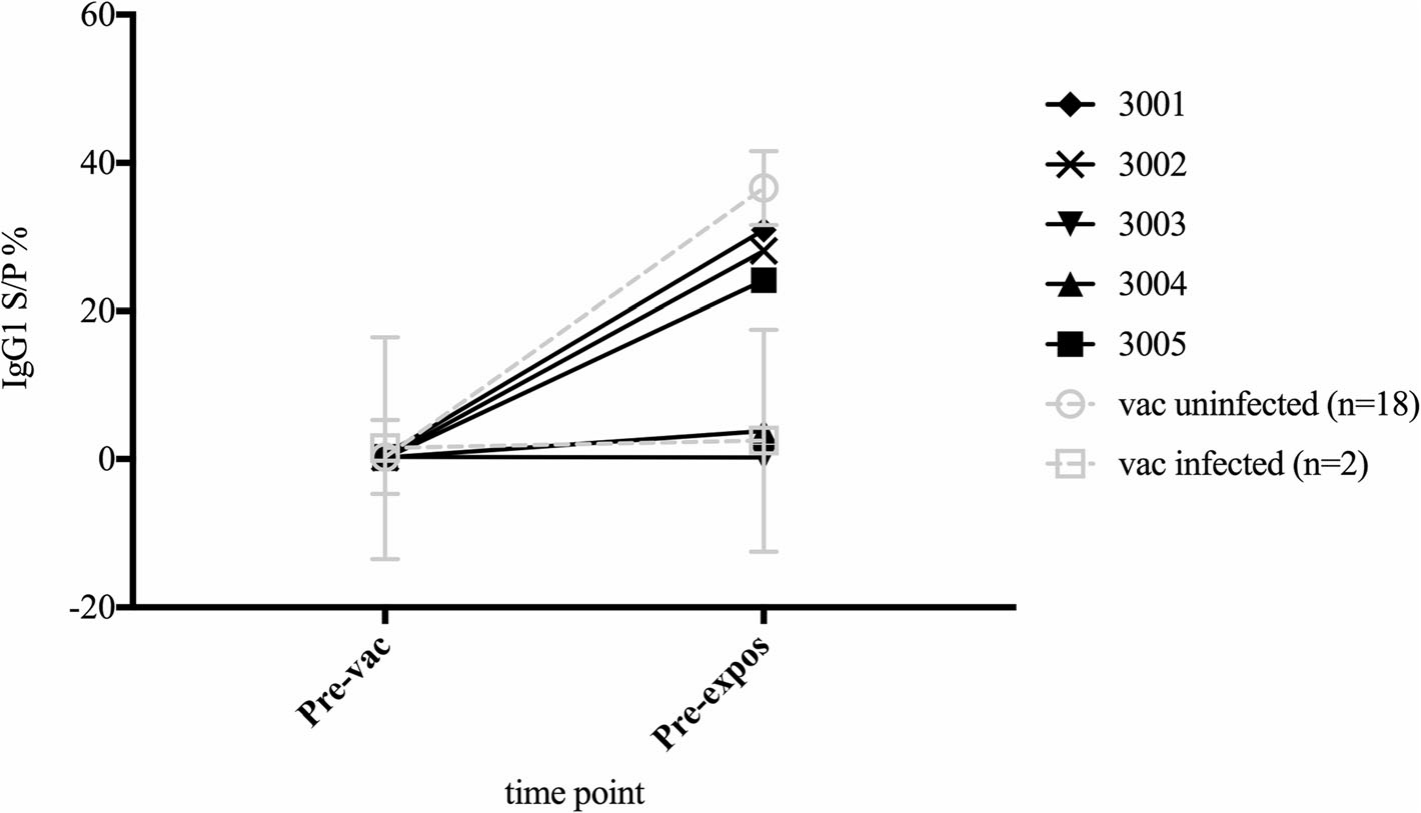
MAP specific-IgG1 serum antibody levels in vaccinated sheep prior to MAP exposure. MAP-specific serum IgG1 levels in vaccinated unexposed sheep (black lines, numbers are individual sheep identifiers) were examined at pre-vaccination and pre-exposure (4 weeks post-vaccination, 2 weeks prior to MAP exposure) time points. The group average values for the vaccinated exposed sheep, categorised as vaccinated infected (tissue culture positive) (*n* = 2) and vaccinated uninfected (tissue culture negative) (*n* = 18) are also shown (dashed grey lines)

The polarised pre-exposure responses seen in the vaccinated infected and uninfected sheep was mimicked in the IgG1 response of vaccinated control animals. The vaccinated control sheep could be differentiated into high and low IgG1 responder groups at the pre-exposure time point (Fig. 3.).

### Gene expression in the ileum

Gene expression analysis of sheep ileal tissue was conducted to assess functionality of B cells at the site of predilection for MAP infection. Fold changes were determined in comparison to a baseline group established from specific research questions (Table 2). This method of analysis was adopted to ensure that the correct controls (baseline groups) were being used to understand the impacts of vaccination, exposure and the spectrum of disease.

**Table 2:** Analysis matrix for sheep B cell-related gene expression in intestinal tissue for specific research questions

Research question	Baseline group	Comparison group (s)
Q1. What gene expression changes are associated with disease outcome in non-vaccinated animals? Are these similar in vaccinated animals?	Non-vaccinated control	Non-vaccinated infected Non-vaccinated uninfected Vaccinated infected Vaccinated uninfected
Q2. What gene expression changes are associated with disease outcome in vaccinated animals	Vaccinated control	Vaccinated infected Vaccinated uninfected
Q3. What gene expression changes are associated with infection in vaccinated animals	Vaccinated uninfected	Vaccinated infected
Q4. What gene expression changes are associated with vaccination nonresponse in infected animals	Non-vaccinated infected	Vaccinated infected

In relation to research question 1, gene expression responses were divergent in vaccinated and non-vaccinated animals that were uninfected at necropsy (Table 3). The majority of genes were down-regulated in the vaccinated uninfected animals, apart from *Lyn* and *NFIL3*. In contrast, in the non-vaccinated uninfected animals only 4 of the 11 genes examined were down-regulated. This dissimilarity of response was also seen in the vaccinated infected and the non-vaccinated infected animals, where only *ERG1* and *GRB2* were down regulated in the non-vaccinated infected, but 4 of the 11 genes were down regulated in the vaccinated infected. Interestingly, vaccinated uninfected animals had significantly decreased expression of *CD84* (fold change 0.112, CI 0.021–0.605*, p* < 0.05) and *BAFF* (fold change 0.06, CI 0.004–0.89*, p* < 0.05) compared to non-vaccinated infected sheep. These same trends were seen in *MIF* expression, with an increase in expression in non-vaccinated infected animals and decreased expression in vaccinated controls and vaccinated uninfected sheep.

**Table 3:** Gene expression changes (fold change) in the ileum of the different treatment groups compared to non-vaccinated control sheep

		Non-vaccinated uninfected (*n* = 10)	Non-vaccinated infected (*n* = 10)	Vaccinated control (*n* = 5)	Vaccinated uninfected (*n* = 18)	Vaccinated infected (*n* = 2)
	^a^IgG1 (SP%)	0.49	10.75	54.39	91.09	52.2
Up regulated	LYN	2.1	1.2		1.4	1.6
	GRB2	1.8				1.4
	ERG1					
	JUN		1.4			1.1
	NFIL3		2.2		1.4	2.8
	LCK	1.5	3.2			
	CD81	1.5	1.9			
	CD84		**3.8** ^**a**^			
	CD40LG	2.5	5			5
	BAFF	1.5	**5** ^**b**^			2.2
	MIF	1.1	2.4			1.3
Down regulated	LYN			−1.8		
	GRB2		−1.2	−3	−1.1	
	ERG1	−1.7	−3.8	−3.3	−1.4	−1.7
	JUN	−1.4		−1.9	− 1.1	
	NFIL3	−1.1		−1.6		
	LCK			−2.8	−1	− 1.1
	CD81			−3.6	−1.6	− 1.2
	CD84	−1.1		−2.1	**−2.4** ^**a**^	−1.1
	CD40LG			−1.1	− 1	
	BAFF			−1.2	**-1** ^**b**^	
	MIF			−2.9	−1.7	

^a^IgG1 SP% of each group at the same timepoint as gene expression analysis was performed. Significant differences between groups are indicated by bolding (*p* < 0.05) with a significantly different to a and b significantly different to b

In research question 2, only the vaccinated animals were examined due to their dissimilarity in expression responses from non-vaccinated sheep. MAP exposure in vaccinated sheep was associated with upregulation of the majority of the genes of interest (Table 4). *CD84* was the only differentially regulated gene, where vaccinated uninfected sheep had decreased expression compared to vaccinated infected sheep. The differences between vaccinated infected and uninfected sheep were further explored in research question 3. The majority of the genes examined were up-regulated in the vaccinated infected sheep compared to the vaccinated uninfected sheep. This increased expression was strongest for *CD40LG*, *BAFF*, *MIF*, *CD84* and *NFIL3* (Table 5). In contrast, both *EGR1* and *Lck* tended to have reduced expression in the vaccinated infected animals compared to the vaccinated uninfected sheep.

**Table 4:** Gene expression changes (fold change) in the ileum of the vaccinated infected and uninfected sheep compared to vaccinated control sheep

		Vaccinated uninfected (*n* = 18)	Vaccinated infected (*n* = 2)
	I^a^gG1 (SP%)	91.09	52.2
Up regulated	LYN	2.56	2.86
	GRB2	2.65	4.33
	ERG1	2.41	1.89
	JUN	1.75	1.99
	NFIL3	2.21	4.47
	LCK	2.72	2.63
	CD81	2.29	3.14
	CD84		1.84
	CD40LG	1.05	5
	BAFF	1.13	2.52
	MIF	1.67	3.82
Down regulated	LYN		
	GRB2		
	ERG1		
	JUN		
	NFIL3		
	LCK		
	CD81		
	CD84	−1.12	
	CD40LG		
	BAFF		
	MIF		

^a^IgG1 SP% of each group at the same timepoint as gene expression analysis was performed

**Table 5:** Gene expression changes (fold change) in the ileum of sheep to answer specific research questions

		Vaccinated infected compared to vaccinated uninfected (Q3)	Vaccinated infected compared to non-vaccinated infected (Q4)
Up regulated	LYN	1.12	1.36
	GRB2	1.64	1.69
	ERG1		2.17
	JUN	1.14	
	NFIL3	2.03	1.28
	LCK		
	CD81	1.37	
	CD84	2.07	
	CD40LG	5.37	
	BAFF	2.22	
	MIF	2.29	
Down regulated	LYN		
	GRB2		
	ERG1	−1.27	
	JUN		−1.36
	NFIL3		
	LCK	−1.03	−3.37
	CD81		−2.17
	CD84		−4.31
	CD40LG		−1.68
	BAFF		−7.52
	MIF		−1.82

In research question 4, the differences in gene expression between non-vaccinated and vaccinated sheep that were infected at necropsy was explored in more detail. The majority of the genes of interest were down regulated in the vaccinated infected animals compared to the non-vaccinated infected sheep (Q4 Table 2). This decreased expression was especially evident in *BAFF*, *CD84* and *Lck* (Table 5).

## Discussion

The exact role of B cells and antibodies in host immunity against intracellular pathogens has been a long-debated topic. We show here that B cell functionality is important in vaccine-induced clearance of infection in animals exposed to MAP. A rapid and potent IgG1 antibody response was seen in all uninfected vaccinated sheep. Furthermore, at 12 months post exposure, these uninfected animals had a dampening of the humoral response at the site of paratuberculosis predilection, in contrast to the late switch to a humoral dominated response often associated with the transition to clinical paratuberculosis. On the other hand, vaccinated infected sheep had a slower IgG1 response to vaccination and had up regulated expression of genes related to the humoral immune response in the ileum at 12 months post exposure.

The serum MAP-specific IgG1 response in vaccinated uninfected sheep was elevated compared to the vaccinated controls and vaccinated infected sheep. This significant difference was already evident prior to MAP exposure suggesting a failure of the host to effectively mount an early IgG1 response post vaccination. The route of entry of MAP into the host macrophage can impact the ability of the host cell to kill it [31, 32]. Opsonised bacteria are more likely to interact with the Fc receptor (FcR) on the surface of monocytes and macrophages [33]. Phagocytosis of opsonised MAP via the FcR increases trafficking of intracellular bacteria to the lysosome and increases killing [33–36]. IgG1 has the highest affinity for the Fc receptor out of all IgG subclasses [37], and it stands to reason that animals that have a high IgG1 response to vaccination would be better equipped to eliminate MAP from the gut [19, 38]. The polarised IgG1 response in the vaccinated control animals, suggests that MAP specific IgG1 levels after vaccination, even in animals without MAP exposure, could be a useful predictor of vaccine efficacy.

The initial low level of serum MAP-specific IgG1 in vaccinated infected sheep, followed by a sharp increase, could signify the change from a Th1 to Th2 mediated response. The switch from a Th1 to Th2 immune response in paratuberculosis has been associated with progression to clinical disease [5]. It is possible that the lack of an early antibody response in these animals aids early tissue invasion and leads to persistent infection, as animals with an inadequate IgG1 response appeared to be incapable of eliminating or controlling MAP. A similar late increase was also seen in the non-vaccinated infected animals, although to a much smaller magnitude. In stark contrast to these two groups, the vaccinated control and vaccinated uninfected animals appeared to have a gradual reduction in MAP-specific IgG1 in the serum towards the end of the trial. This reduction could signify a waning of the initial strong antibody response, either due to clearance of bacteria and decreased chronic antigenic stimulation, or the return to a more balanced immune state. The reduction of antibody levels, and presumably B cell response, could also lead to reductions in B cell related immunopathologies associated with an excessive response [39].

In the non-vaccinated sheep, there was no significant difference in levels of either IgG or IgG1 between the control, infected or uninfected animals at any sampling point. Low levels of MAP-specific IgG and IgG1 antibody are not unexpected in MAP exposed animals during the early stages of disease [4]. The differences seen between the vaccinated and non-vaccinated uninfected animals in terms of MAP-specific IgG1 could be the result of differences in the stimulus to induce protective immune responses. The immune response generated by a vaccine such as Gudair® is much stronger than that generated by natural exposure to MAP, due to continual antigen presentation via depot formation and the immune enhancing properties of the adjuvant [40].

The comparison of gene expression changes in the gut of sheep from multiple treatment groups created a dilemma as to what makes the best baseline group to determine fold change. As such, several different research questions were presented to explore the effects of vaccination, exposure to MAP and infection status on the expression of B cell related genes in the gut of ruminants.

Initially, to examine the impacts of both vaccination and infection outcome, the non-vaccinated control sheep were used as the baseline (Q1 Table 2). Vaccination alone decreased B cell-related gene expression and a similar pattern was found in vaccinated animals that resisted or recovered from infection (vaccinated uninfected). Previous work has suggested that early loss of B cell functionality could contribute to vaccine non-response [16], this lack of B cell activation in the current study could be due to samples being taken at a much later time point as well as differences in sample type (blood vs ileal tissue). There was an up-regulation of genes in ileal tissues in infected animals (both vaccinated and non-vaccinated) compared to healthy controls. Overall the results from this study suggests that a B cell response in the later stages of disease, at the site of infection, is not beneficial. Similarly, to the serum antibody results, a strong initial B cell mediated response could be beneficial, whilst a late response signifies progression to disease. The pattern of reduced expression in the vaccinated uninfected animals is likely to be due to clearance of MAP and subsequent return to an inactive or homeostatic immune state, which would be a response similar to the vaccinated unexposed animals where there is also no MAP to activate expression of B cell related genes.

In vaccinated animals, increased expression of CD40 ligand (*CD40LG*), *JUN* and B cell activating factor (*BAFF*) were related to infection status (Q2 Table 2). Engagement of CD40LG with the CD40 receptor on B cells is required for initiation and maintenance of the humoral immune response [41]. In the initial stages of humoral immunity after exposure, CD40/CD40LG signalling is required for the generation of high titres of class switched, high affinity antibodies [41]. During the progression of the immune response, signalling through these receptors encourages the development of memory B cells [39, 41]. The ligation of CD40 has been associated with production of AP-1 early response transcription factor, which is a heterodimer of cFOS and cJUN [42]. Overexpression of cJUN has been associated with prevention of apoptosis, highly increased proliferation and even immortalisation of B cells [43]. BAFF also plays a pivotal role in promoting the survival of plasmablasts, especially after CD40/CD40LG activation [39]. Proliferation and class switch triggered by CD40/CD40LG and then the prevention of apoptosis by BAFF and AP-1 would push the host’s immune response to one that is Th2 dominated [44]. The reduced expression of this gene in the vaccinated uninfected animals suggests that, at this timepoint, the humoral response in these animals is being down regulated or suppressed.

Interestingly, only *Lck* and *EGR1* were down-regulated in the vaccinated infected sheep compared to the vaccinated uninfected animals, although this was not statistically significant. The expression of both of these genes influences B cell maturation, differentiation and antigen receptor signalling [45, 46]. Host tyrosine kinases, like *Lck*, are key host molecules utilised by intracellular pathogens to prevent killing [47]. In agreement with our work, the suppression of members of the *Src* family in chicken B cells renders them largely unresponsive to stimulation through the antigen receptor [45]. *EGR1* expression is induced upon B cell antigen receptor signalling and accelerates B cell maturation [46]. The dysfunction of the B cell antigen receptor as a possible result of *Lck* gene down regulation could result in the reduced expression of *EGR1*. Therefore, it is possible that either upon vaccination or following MAP exposure B cell maturation and differentiation has been reduced through the supressed expression of these two genes, which is likely to diminish the memory response and could contribute to MAP persistence.

Although statistically significant differences were seen in this study, only a small number of the vaccinated exposed animals were infected at necropsy (*n* = 2). To validate the findings of this research, a larger number of vaccinated infected animals should be examined.

## Conclusions

In conclusion, B cell responses were shown to be important to vaccine-mediated immune protection. A strong initial B cell response, characterised by MAP-specific IgG1 levels in serum, was seen in vaccinated sheep that cleared infection. Furthermore, this response appears to be toned down or tightly regulated towards the later stages of infection to prevent the dominance of the humoral response that likely marks the progression to clinical disease. The unique insight into the mechanisms behind vaccine immunity provided by this study will allow vaccine development to promote a strong initial humoral response and could possibly contribute to genetic selection for vaccine response in the future.

## Methods

### Animal trial

Fifty Merino sheep were purchased from a farm participating in the Australian Market Assurance scheme for Paratuberculosis. The wether lambs aged 4 months were sourced from a flock in Armidale, New South Wales (NSW), an area that has no prior history of Johne’s disease (JD). Absence of MAP infection was confirmed through extensive whole flock faecal tests and serum antibody ELISA [48]. On arrival at the university farm in Camden, NSW, sheep were randomly allocated into 4 treatment groups, Gudair™ vaccinated MAP unexposed (*n* = 5) and exposed (*n* = 20), Non-vaccinated MAP unexposed (*n* = 5) and exposed (*n* = 20). Sheep were managed under conventional Australian sheep farming conditions by grazing in open paddocks on unimproved pasture. During the trial all animals were examined daily and were weighed monthly. Faecal samples were collected from the rectum and blood samples via jugular venepuncture of all animals prior to inoculation and then every 3–4 months; collection order was non-purposive by selecting animals as they entered the handling yards. Blood and faecal samples were collected in sheep handling yards within the university farm land, and undertaken at a time of day when the weather was mild (between 15 and 25 °C) to reduce stress to the animals.

Vaccination with Gudair® was performed in accordance with the manufacturer’s instructions and at 6 weeks post-vaccination a cohort were moved to quarantine paddocks and inoculated with MAP (Telford 9.2). The inoculation consisted of 3 oral doses (within a 4-week period) as described by Begg et al. [48] with a total of 9.25 × 10^8^ viable MAP organisms. A non-vaccinated cohort was similarly exposed to MAP at the same time. Unexposed animals, vaccinated and non-vaccinated, were kept in separate paddocks to their exposed counterparts.

At the conclusion of the trial (52wpe), all animals were euthanised using an intravenous injection of barbiturate (Lethabarb™) at 0.5 ml/kg bodyweight. The disease status of all MAP exposed animals was then categorised based on liquid culture of MAP from gut tissues collected at necropsy, as described previously [49–51]. Animals with positive tissue cultures were classified as infected and those with negative results were classified as uninfected. A smaller subset of animals was used for gene expression examination in gut tissue (Table 1).

### Serum antibody levels

#### MAP specific IgG1 ELISA

ELISA plates (Nunc Maxisorb) were coated with 5 μg/ml MAP 316v, Protoplasmic antigen A (PPA) or heat-killed *M. phlei*. The ELISA plates were machine washed 5 times (Tecan, Austria) using wash buffer (phosphate buffered saline with 0.05% v/v Tween 20). Diluted serum (1/100) was added in duplicate to each antigen. Plates were incubated at 37 °C for 1 h, washed as described above and anti-IgG1 antibody (AbD Serotec, MCA 2440) was added. After a 1-h incubation at 37 °C, plates were machine-washed 5 times. Goat anti-mouse HRP (Dako P0447) was then added to each well and incubated at 37 °C for 30 min. Plates were washed as described earlier and TMB substrate (Pierce) added prior to incubating at room temperature in the dark for 20 min, the reaction was stopped with 2 M sulphuric acid and plates were read at 450 nm.

A single batch of positive and negative controls were included on each plate to standardise the ELISA. The positive serum controls were sourced from a sheep with high MAP specific serum antibody levels, as identified by the commercial IDEXX ELISA and the IgG1 ELISA. The negative control was serum from a sheep consistently test-negative for MAP-specific antibodies, as determined by the IDEXX ELISA and the IgG1 ELISA.

The MAP 316v antigen-specific IgG1 response was calculated using the following formula:

$$ SP\%=\frac{OD_{sample}-{OD}_{PPA\  negative\ control}}{OD_{PPA\  positive\ control}-{OD}_{PPA\  negative\ control}}\times 100 $$

*M. phlei* was included as a mycobacterial cross-reactivity control to ensure that responses seen in the IgG1 ELISA were MAP specific. PPA was included in the plate as a second MAP specific antigen and the ratio of PPA and 316 V response (1:0.8) was used to monitor positive control performance. Furthermore, the stronger PPA response was utilised to calculate SP%.

#### MAP specific IgG ELISA

The commercial IDEXX Pourquier ELISA (Idexx Laboratories, Australia) was used to determine MAP-specific serum IgG antibody levels. The ELISA was performed based on the manufacturer’s instructions.

### Faecal MAP detection

A high throughput direct faecal PCR was used to quantify the amount of MAP shed in the faeces of all sheep, as previously described [52].

### Gene expression at the site of infection

#### Gene selection

The genes to be examined were selected from a normalised and statistically analysed data set generated from previous microarray gene expression analysis (Affymetrix GeneChip) on sheep peripheral blood mononuclear cells (PBMC). This data set was generated from two sheep experimental infection trials that compared differential gene expression in animals vaccinated with Gudair™ and experimentally exposed to MAP compared to non-vaccinated MAP-exposed animals. Samples for gene expression analysis were taken at 13 wpe and 18 animals were used (9 vaccinated infected and 9 vaccinated uninfected). The raw data was normalised using the RMA (Robust Multichip Averaging) algorithm and significant differences were determined with ANOVA [53].

The data set was then examined using Ingenuity Pathway Analysis (IPA) software (version 01–01, Qiagen Bioinformatics). IPA was used to search for genes related to B cell functionality, survival, receptor signalling, migration and interaction with other immune cells within the data set. The overlay function was then used to examine the gene expression fold change in the microarray data set in relation to vaccination and disease outcome. Genes for qPCR analysis for the current study (Table 6) were then selected based on a fold change of greater than +/− 1.5 in PBMC.

**Table 6:** Selected genes and primers used for gene expression analysis of intestinal tissue

Gene name	Entrez gene ID	Primer sequence	Tm	Product size (bp)	Gene Function
LYN	100,302,103	5′-ACGGAGAGTGGTGGAAAGC-3′ 5′-GTGCACGGGGTCATAGTCT-3’	59.6 59.1	482	B cell signaling + activation
GRB2	101,109,893	5’-ACTGCTGCTCCTGTTCTTCC-3′ 5′-AAACGCAGAACACAGAAGCG-3’	60.0 59.7	442	B cell signal transduction + communication
EGR1	443,547	5’-CCCCGACTATCTGTTTCCACA-3′ 5′-ATGCGGCTGGGTTTGATGA-3’	59.5 60.0	340	B cell maturation + activating factor signaling
JUN	443,219	5’-CAAGTGCCGGAAAAGGAAGC-3′ 5′-ACAGTCTCGCCTCAAAACGT-3’	59.5 59.8	314	Transcription regulator
NFIL3	100,217,409	5’-CACTGTGAGCGCCTTTGTG-3′ 5′-GGGCCCTCCTGTGAATGTT-3’	59.7 59.6	262	B Cell survival
LCK	100,216,439	5’-CCCAGCTTCTCCACTGCAA-3′ 5′-ACGGAGCTGTTCACCCTTC-3’	59.9 59.6	270	B cell activation
CD81	100,147,790	5’-CTGGGCACGTTCTTCACCT-3′ 5′-GCTGCAAGGCCTGGTCATA-3’	59.3 60.0	479	B cell development, activation, growth and motility
CD84	101,102,779	5’-CGTGGAACCCTGTCAGCAA-3′ 5′-AGCACAGAAAGCACAGCCA-3’	60.2 60.2	412	Found on memory B cells
CD40L	767,628	5’-AGCTGGCCGTGAAAAGACA-3′ 5′-AACACCGAAGCACCCGATT-3’	59.9 59.9	489	Initiation and maintenance of humoral immune response
BAFF (TNFSF13B)	101,104,901	5’-TTGCAGACAGTGACACGCC-3′ 5′-AGGTGTCCCATGGCAAAGG-3’	60.9 59.9	426	B Cell survival
MIF	780,466	5’-GCAAGCCGGCACAGTACAT-3′ 5′-ATGTAGATCCTGTCCGGGCT-3’	61.0 60.1	305	Survival of mature B cells + NF-kB signalling cascade
Reference gene					
Ovine GAPDH	443,005	5’-AGAAACCTGCCAAGTATGATG-3′ 5′-CCTAGAATGCCCTTGAGAGG-3’	60.5 62.6	76	

#### Tissue sections

At the conclusion of the trial (52 wpe), all animals were necropsied. The gastrointestinal tract was removed and 3–4 cm sections of the mid to terminal ileum were excised. The sections were frozen at − 80 °C prior to RNA extraction.

#### RNA extraction and quality and quantity assessment

RNA extraction was performed using RNAzol® RT (Merc) per the manufacturer’s instructions. The quality and quantity of purified RNA was assessed using a NanoDrop® ND-1000 UV-Vis Spectrophotometer (Thermo Scientific, Wilmington, DE), using the Nucleic Acid module. The absorbance at 260 nm was used to determine the RNA concentration where an A_260_ nm reading of 1.0 is equivalent to 40 μg/mL of RNA. Purity was characterised as a A_260_/A_280_ ratio between 1.8 and 2 and a A_260_/A_230_ ratio between 1.4 to 2.2. To remove contaminating genomic DNA and increase RNA purity, the samples were DNase treated and ethanol precipitated following extraction.

#### cDNA generation

cDNA was generated from RNA using the iScript™ cDNA Synthesis kit (Bio-Rad) per the manufacturer’s instructions, diluted 1/10 and stored at − 80 °C until required.

#### Primer selection and validation

Forward and reverse primers (Table 6) were designed specifically for the gene regions of interest using online software Primer 3 [54] and checked for specificity using a BLAST search. As genes were selected based on expression levels in PBMC, gene expression in intestinal tissues was confirmed using cDNA generated from a paratuberculosis infected sheep.

Three additional housekeeping genes were assessed with geNorm analysis in the qBASE plus analysis software (Biogazelle) [55]. This analysis identified the most stable reference; for subsequent analyses one reference gene was used based on the geNorm analysis (Table 5).

#### qPCR and gene expression level analysis

qPCR was performed using an Mx3000P Real-time PCR system (Stratagene, Agilent) using the SensiMix™ SYBR® kit (Bioline). Assays were prepared in 96 well plates and included duplicates of each sample. Reaction volumes of 25 μl (including 10 μl of target cDNA at a 1/10 dilution) were prepared and amplified under the following conditions: 95 °C for 10 min, then 40 cycles of 95 °C for 20 s, 56 °C for 30 s and 72 °C for 30 s, with fluorescence acquisition at the end of each annealing step. The specificity of the reaction was confirmed using melting curve analysis. Standard curves were performed on each plate for each primer set. Data collected from the quantitative reverse transcription (qRT)-PCR were analysed using qBASE plus analysis software (Biogazelle) utilising a modified Comparative Ct (ΔΔCt) method [56]. Fold changes were determined in comparison to pre-selected baseline group (Table 2) and the biological significance was set at a change of +/− 1.5 fold.

### Statistical analysis

Restricted maximum likelihood (REML) in a linear mixed model (Genstat 16th edition; VSN International Ltd., Hemel Hempstead, United Kingdom) was used to analyse the MAP-specific IgG1, IgG serum ELISA results (S/P %) and MAP DNA quantity in the faeces. Sheep were grouped based on treatment coupled with infection status (vaccinated control, vaccinated infected, vaccinated uninfected, non-vaccinated control, non-vaccinated infected or non-vaccinated uninfected), which along with sampling time point was included as a fixed effect in the model. Animal ear tag number was included as a random effect. When the REML analysis was significant, post-hoc tests to determine the significant differences between pairs of predicted means using the Fisher’s Least Significant Difference procedure were performed.

## Data Availability

The datasets used and/or analysed during the current study available from the corresponding author on reasonable request.

## Abbreviations

BAFF: B cell activating factor
CD40L: CD40 ligand
CMI: Cell mediated immunity
ELISA: Enzyme linked immunosorbent assay
FcR: Fc receptor
IFNγ: Interferon gamma
IPA: Ingenuity pathway analysis
LcK: Lymphocyte specific protein kinase
MAP: *Mycobacterium avium* subspecies *paratuberculosis*
MIF: Macrophage migration inhibitory factor
PBMC: Peripheral blood mononuclear cell
PCR: Polymerase chain reaction
PPA: Protoplasmic antigen A
REML: Restricted maximum likelihood
wpe: Weeks post exposure
